# Differential Involvement of Kinase Activity of Ca^2+^/Calmodulin-Dependent Protein Kinase IIα in Hippocampus- and Amygdala-Dependent Memory Revealed by Kinase-Dead Knock-In Mouse

**DOI:** 10.1523/ENEURO.0133-18.2018

**Published:** 2018-08-21

**Authors:** Yoko Yamagata, Yuchio Yanagawa, Keiji Imoto

**Affiliations:** 1 Division of Neural Signaling, National Institute for Physiological Sciences, Okazaki 444-8787, Japan; 2 SOKENDAI (The Graduate University for Advanced Studies), Okazaki 444-8787, Japan; 3 Department of Genetic and Behavioral Neuroscience, Gunma University Graduate School of Medicine, Maebashi 371-8511, Japan

**Keywords:** calmodulin kinase II, fear conditioning, knock-in mouse, phosphorylation, PTSD, water maze

## Abstract

Ca^2+^/calmodulin-dependent protein kinase IIα (CaMKIIα) is a key mediator of activity-dependent neuronal modifications and has been implicated in the molecular mechanisms of learning and memory. Indeed, several types of CaMKIIα knock-in (KI) and knock-out (KO) mice revealed impairments in hippocampal synaptic plasticity and behavioral learning. On the other hand, a similar role for CaMKIIα has been implicated in amygdala-dependent memory, but detailed analyses have not much been performed yet. To better understand its involvement in amygdala-dependent memory as compared to hippocampus-dependent memory, here we performed biochemical analyses and behavioral memory tests using the kinase-dead CaMKIIα (K42R)-KI mouse. In the Morris water maze tasks, homozygous mutants performed well in the visible platform trials, while they failed to form spatial memory in the hippocampus-dependent hidden platform trials. In fear conditioning, these mice were impaired but showed a certain level of amygdala-dependent cued fear memory, which lasted four weeks, while they showed virtually no hippocampus-dependent context discrimination. Neither stronger stimulation nor repetitive stimulation compensated for their memory deficits. The differential outcome of hippocampus- and amygdala-dependent memory in the mutant mouse was not due to differential expression of CaMKIIα between the hippocampus and the amygdala, because biochemical analyses revealed that both kinase activity and protein levels of CaMKII were indistinguishable between the two brain regions. These results indicate that kinase activity of CaMKIIα is indispensable for hippocampus-dependent memory, but not necessarily for amygdala-dependent memory. There may be a secondary, CaMKIIα activity-independent pathway, in addition to the CaMKIIα activity-dependent pathway, in the acquisition of amygdala-dependent memory.

## Significance Statement

Studying molecular mechanisms of learning and memory is important to confront memory-deficient and abnormal memory-associated disorders. An enzyme called Ca^2+^/calmodulin-dependent protein kinase IIα (CaMKIIα) that is abundant in the brain and phosphorylates important proteins has a key role in such mechanisms. However, how CaMKIIα enzymatic activity is involved in hippocampus- versus amygdala-dependent memory is still not clear. Using our genetically engineered mouse that lacks kinase activity but retains protein expression of CaMKIIα, here we showed that kinase activity of CaMKIIα is indispensable for hippocampus-dependent space/context-related memory, but not necessarily for amygdala-dependent fear-related memory. The role of CaMKIIα kinase activity in distinguishing different contexts indicates its possible involvement as a measure against abnormal fear memory-associated disorders, such as posttraumatic stress disorder (PTSD).

## Introduction

Ca^2+^/calmodulin-dependent protein kinase II (CaMKII) is one of the most abundant protein kinases in the central nervous system, and is thought to be a key mediator of activity-dependent neuronal modifications (Hudmon and Schulman, 2002; Lisman et al., 2002, 2012; Coultrap and Bayer, 2012; Hell, 2014; Herring and Nicoll, 2016a; Takemoto-Kimura et al., 2017). The neuronal CaMKII holoenzyme is assembled from homologous 12 subunits consisting of CaMKIIα and/or CaMKIIβ, the two major isoforms in the brain, with CaMKIIα dominant in the forebrain, and with CaMKIIβ dominant in the cerebellum. CaMKIIα is especially enriched in the hippocampus, and has been proposed to be the indispensable isoform to hippocampal synaptic plasticity and behavioral learning (Lisman et al., 2002; Lisman, 2017; Rossetti et al., 2017; Takemoto-Kimura et al., 2017).

Activity-dependent Ca^2+^ influx into neurons causes activation of CaMKII by binding of Ca^2+^/calmodulin. Activated CaMKII translocates to the postsynaptic sites and undergoes T286(α)/T287(β)-autophosphorylation within the autoinhibitory region through an intersubunit mechanism. This autophosphorylation makes the kinase persistently active and prolongs its postsynaptic association even after intracellular Ca^2+^ concentration is reduced. There, the kinase phosphorylates a number of substrates including AMPA-type glutamate receptors (AMPARs), transmembrane AMPAR regulatory proteins, synaptic Ras-GTPase activating protein, and Rho guanine nucleotide exchange factors (Lee et al., 2003, 2010; Xie et al., 2007; Yang et al., 2013; Araki et al., 2015; Herring and Nicoll, 2016b; Park et al., 2016), which leads to long-term potentiation (LTP) of the synapse, one of the fundamental mechanisms for learning and memory. When intracellular Ca^2+^ concentration is reduced, Ca^2+^/calmodulin is detached from CaMKII, and another autophosphorylation at T305/T306(α)/T306/T307(β) within the calmodulin-binding region occurs through an intrasubunit mechanism. This autophosphorylation prevents rebinding of Ca^2+^/calmodulin, reduces kinase activity to about a half and facilitates its dissociation from postsynaptic sites to terminate its action at the synapse.

So far, a number of genetically engineered CaMKIIα knock-out (KO) and knock-in (KI) mice confirmed the direct involvement of CaMKIIα in LTP, and learning and memory (Silva et al., 1992; Giese et al., 1998; Elgersma et al., 2002; Yamagata et al., 2009; Achterberg et al., 2014). Among them, functionally modified KI mice, especially the CaMKIIα (T286A)-, (T305D)-, and (K42R)-KI mice showed severe deficits not only in hippocampal LTP, but also in hippocampus-dependent memory, revealing the importance of persistent activation, postsynaptic association and enzymatic activity of CaMKIIα, respectively, in such processes (Giese et al., 1998; Elgersma et al., 2002; Yamagata et al., 2009). Thus, functionally intact CaMKIIα is critically involved in the acquisition of hippocampus-dependent memory. A recent study using the region-specific and time-specific conditional CaMKIIα KO mice also confirmed its requirement in the forebrain and at the time of learning (Achterberg, et al., 2014).

On the other hand, how CaMKIIα is involved in other types of memory is still not clear. Previous studies using fear conditioning in rats (Rodrigues et al., 2004) and in the CaMKIIα (T286A)-KI mouse (Irvine et al., 2005, 2011) indicated a similar involvement of CaMKIIα in amygdala-dependent memory as in hippocampus-dependent memory. A recent study using a light-inducible CaMKII inhibitor also supported a role for CaMKII activity in the amygdala in the acquisition of inhibitory avoidance memory (Murakoshi et al., 2017). However, detailed analyses have not much been performed yet using other types of CaMKIIα mutant mice.

We previously generated the kinase-dead CaMKIIα (K42R)-KI mouse to examine the specific role of kinase activity of CaMKIIα, separately from its other protein functions (Yamagata et al., 2009). In this mouse, kinase-dead CaMKIIα (K42R) could translocate to postsynaptic sites in response to synaptic activation, whereas tetanic stimulation could not induce LTP or dendritic spine enlargement in the hippocampus. Besides, inhibitory avoidance memory was severely impaired. Thus, we could show that kinase activity of CaMKIIα is essential for hippocampal synaptic plasticity and behavioral learning. Here we took the advantage of this KI mouse to examine how kinase activity of CaMKIIα is involved in amygdala-dependent memory, as compared to hippocampus-dependent memory, to see if there is any mechanistic difference between them.

## Materials and Methods

### Animal experiments

The kinase-dead CaMKIIα (K42R)-KI mouse (B6.Cg-*Camk2a^tm1.1Oyam^*) was generated as previously described (Yamagata et al., 2009; RBRC05821, RIKEN BRC). All animal experiments were reviewed and approved by the Institutional Animal Care and Use Committee of National Institutes of Natural Sciences. All experiments were conducted in accordance with the Guide for Animal Experimentation in the Institute. Animals were housed in cages with ad libitum access to water and food and maintained on a 12/12 h light/dark cycle.

All analyses were performed using adult homozygous CaMKIIα (K42R)-KI and wild-type littermate control mice generated by intercrosses between heterozygous mice backcrossed to C57BL/6 for more than six generations. Male or female mice were used for biochemical analyses, and male mice were used for behavioral analyses. Mice that showed any signs of seizure were excluded from the experiments (Yamagata et al., 2009).

### Sample preparation for biochemical analyses

Brain homogenates from the hippocampus and the amygdala were prepared separately as previously described with some modifications (Yamagata et al., 2009). Animals were individually decapitated under carbon dioxide anesthesia, and brains were removed quickly, put in ice-cold homogenization buffer within 30 s after decapitation, and left in the buffer for 30 s for chilling. The hippocampus was dissected on an ice-cold Petri dish. The amygdala was dissected from separate animals as follows: A two-mm thick coronal slice containing most of the amygdaloid complex (0.8–2.8 mm posterior to the bregma) was cut by using an ice-cold Rodent Brain Matrix (ASI Instruments), and its ventro-lateral portion against the striatum and lateral ventricle was cut bilaterally on an ice-cold Petri dish. The remaining slice was checked afterward under a stereomicroscope to verify that the dissected parts corresponded to the amygdaloid complex and the adjacent piriform cortex. Dissected pieces of tissue were immediately frozen in liquid nitrogen and stored at -80°C until use. Frozen pieces of hippocampi (collected from three to five animals) or amygdala (collected from five to seven animals) were homogenized in a five-fold volume of homogenization buffer in a Teflon-glass homogenizer on ice to make an independent experimental sample. The homogenization buffer consisted of 20 mM Tris/HCl, pH 7.5, 5 mM EDTA, 1 mM EGTA, 10 mM sodium pyrophosphate, 50 mM NaF, 1 mM Na_3_VO_4_ (ortho), 1 mM dithiothreitol, 10 μg/ml each of leupeptin, antipain, pepstatin, and chymostatin, 0.1 mM phenylmethylsulfonyl fluoride, and 0.1 μM calyculin A. Each sample was quickly aliquoted, an aliquot was saved for the preparation of samples for SDS-PAGE, another for the measurement of protein concentration, and the rest were frozen immediately and stored at -80°C until additional characterization of kinase activity. Samples for SDS-PAGE were prepared as previously described (Yamagata and Obata, 1998; Yamagata et al., 2009). Protein concentration was determined by using BCA Protein Assay Reagent (Pierce, Thermo Fisher Scientific) and bovine serum albumin as a standard.

### CaMKII kinase activity assay

**Figure 1.**
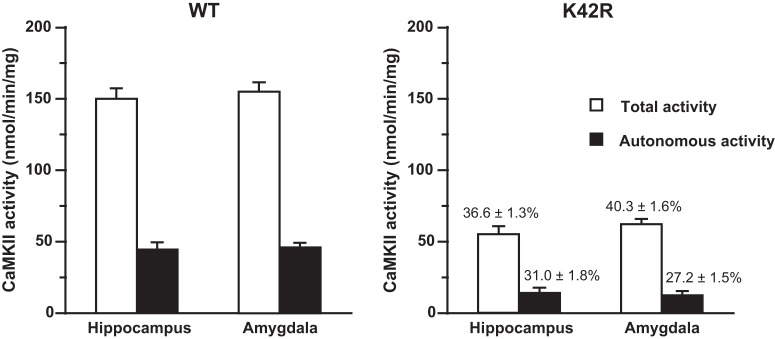
Kinase activity of CaMKII is basically the same between the hippocampus and amygdala in the wild-type mouse and in the kinase-dead CaMKIIα (K42R)-KI mouse. Left, Total and Ca^2+^/calmodulin-independent autonomous activity of CaMKII in homogenates from the hippocampus and amygdala of wild-type (WT) mice. These values reflect the summation of the activity of CaMKIIα and CaMKIIβ, the two major CaMKII isoforms in the brain. Right, Kinase activity of CaMKII in homogenates from the hippocampus and amygdala of kinase-dead homozygous CaMKIIα (K42R)-KI mice. These values reflect the activity of intact CaMKIIβ. Note that figures in percentages above the columns represent relative activity against the value of corresponding control wild-type activity. The total activity was measured in the presence of Ca^2+^/calmodulin. The Ca^2+^/calmodulin-independent autonomous activity was measured in the absence of Ca^2+^/calmodulin. No significant difference was observed in the activity between the hippocampus and amygdala in both genotypes (see text in detail). Open columns, total activity; filled columns, Ca^2+^/calmodulin-independent autonomous activity. Error bars indicate SEM. Hippocampus, *n* = 6; amygdala, *n* = 6 for each genotype.

CaMKII kinase activity assay was performed as previously described (Yamagata et al., 2009). The assay was conducted in the presence of 50 mM HEPES/NaOH, pH 7.5, 10 mM magnesium acetate, 1 mM EGTA, 50 μg/ml BSA, 0.1% Triton X-100, 50 μM autocamtide-2 [KKALRRQETVDAL (Hanson et al., 1989); synthesized by Mimotopes], 2 μM PKI-(5-24)-amide (Peninsula Laboratories), 2 μM PKC-(19-36)-amide (Peninsula Laboratories), 100 μM [γ-^32^P]ATP (400-800 cpm/pmol, PerkinElmer), and with (for the total activity) or without (for the Ca^2+^/calmodulin-independent autonomous activity) 1.5 mM CaCl_2_ and 25 μg/ml calmodulin in a final volume of 50 μl. The reaction was started by the addition of [γ-^32^P]ATP, performed for 1 min at 30°C, and terminated by the addition of acetic acid (final concentration, 10%). After spotting aliquots onto pieces of P81 phosphocellulose paper (Whatman, Thermo Fisher Scientific) and washing the paper with 75 mM phosphoric acid for five times, the retained radioactivity on the paper was measured in a beta scintillation counter (Beckman Coulter). The amount of protein used for the kinase activity assay was 0.6 μg from hippocampal or amygdala homogenates, and the reaction was linear in terms of both protein concentration and incubation time. Kinase activity was expressed as the amount of ATP incorporated into the substrate peptide in 1 min/mg of protein (nmol/min/mg), and compared between the hippocampus and amygdala in each genotype by using unpaired *t* test, two-tailed, *n* = 6 for each brain region from each genotype (Fig. 1). When relative activity of KI samples was calculated, it was expressed as a percentage against the value of control wild-type samples in the same experimental group measured on the same day (Fig. 1, right, above the columns).

### Immunoblot analyses

**Figure 2.**
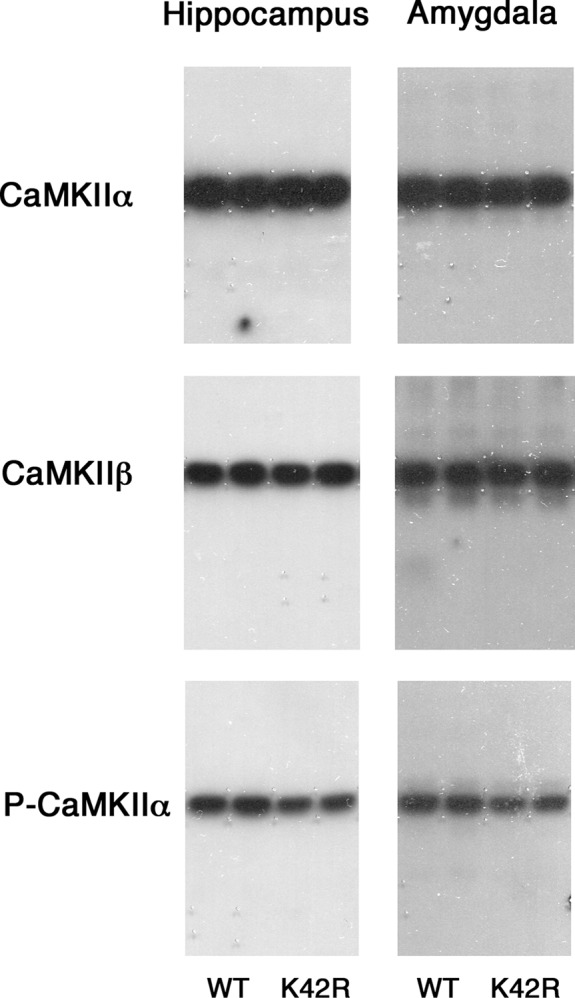
Representative immunoblots showing the CaMKIIα, CaMKIIβ and phospho-T286-CaMKIIα levels in the hippocampus and amygdala of the kinase-dead CaMKIIα (K42R)-KI mouse as compared to the wild-type mouse. The amounts of protein used were 2, 4, and 8 μg for the detection of CaMKIIα, CaMKIIβ, and phospho-T286-CaMKIIα (P-CaMKIIα), respectively, from hippocampal or amygdala homogenates. Autoradiography of duplicated samples from a pair of wild-type (WT) and kinase-dead CaMKIIα (K42R)-KI mice in the same experimental group used for quantitative immunoblot analyses are shown. See also Table 1.

Quantitative immunoblot analyses were performed as previously described (Yamagata et al., 2009). Equal amounts of protein from each sample were subjected to SDS-PAGE, transferred to nitrocellulose transfer membranes (Whatman, Thermo Fisher Scientific), and immunoblotted using one of the following antibodies. Antibodies against CaMKIIα (mouse monoclonal; 6G9; 1:1000; BIOMOL catalog #SA-112, Enzo Life Sciences; RRID: AB_10617228), CaMKIIβ (mouse monoclonal; CB-β-1; 1:200; Zymed catalog #13-9800, Thermo Fisher Scientific; RRID: AB_2533045), and phospho-T286-CaMKIIα (rabbit polyclonal; 1:500; Promega catalog #V1111) were used for the primary reaction. For mouse antibodies, rabbit anti-mouse IgG (1:500; MP Biomedicals catalog #55480) was used in the secondary reaction. Blots were then visualized by using ^125^I-protein A (3.5–5 × 10^5^ cpm/ml; PerkinElmer), and by exposing to an x-ray film, as previously described (Yamagata and Nairn, 2015). The immunoreactive bands were cut out, and their radioactivity was quantitated by using a gamma counter (Hitachi Aloka Medical). The amounts of protein used were 2 μg (for anti-CaMKIIα), 4 μg (for anti-CaMKIIβ), and 8 μg (for anti-phospho-T286-CaMKIIα) from hippocampal or amygdala homogenates. The measured immunoreactivity was in a linear range in terms of protein amounts used for each antibody. The values obtained from KI samples were expressed as percentages against those from control wild-type samples in the same experimental group on the same blots, and analyzed by using one sample *t* test, two-tailed, *n* = 6 for each brain region (Fig. 2; Table 1).

**Table 1.** CaMKIIα and CaMKIIβ protein levels and phospho-T286-CaMKIIα level in the hippocampus and amygdala from the kinase-dead CaMKIIα (K42R)-KI mouse as compared to the wild-type mouse

	Hippocampus (% of WT)	Amygdala (% of WT)
CaMKIIα	85.1 ± 5.1*	81.0 ± 5.1*
CaMKIIβ	101.8 ± 6.7	105.6 ± 6.8
Phospho-T286-CaMKIIα	63.1 ± 6.8**	69.5 ± 3.2***

Significantly different from wild-type (WT) levels: *p < 0.05, **p < 0.01, ***p < 0.001 (one sample *t* test); hippocampus, *n* = 6; amygdala, *n* = 6.

### Animals used for behavioral experiments

Mice were three to five months of age at the start of behavioral experiments. They were accustomed to the experimenter by careful handling for more than one week before the start of experiments. Behavioral experiments were conducted with the experimenter blind to the genotype of mice.

### Water maze

Water maze procedure followed basically as described by Crawley (2000). A circular pool with a diameter of 1 m was filled with opaque water colored with white paint to a depth of 20 cm. Water temperature was maintained at 24–25°C. The escape platform with a diameter of 11 cm was submerged 1 cm below the water surface. The visible cue for the platform consisted of a black pole (10 cm tall) with a ball on top colored black in its lower half (3.5 cm in diameter) standing in the center of the platform. The swimming paths of animals were recorded by a black and white charge-coupled device video camera mounted above the center of the pool using LabVIEW and Vision software (National Instruments; RRID: SCR_014325), and analyzed by Igor Pro 6 software (WaveMetrics; RRID: SCR_000325). Extra-maze cues were posted above the wall of the pool as spatial references.

Naive mice were first accustomed to the water without spatial cues and the visible cue for the platform on the day before the start of water maze training. They were gently released into the pool, allowed to swim for 30 s, then guided onto the submerged platform and allowed to remain there for 30 s. This procedure was repeated three times for each mouse.

**Figure 3.**
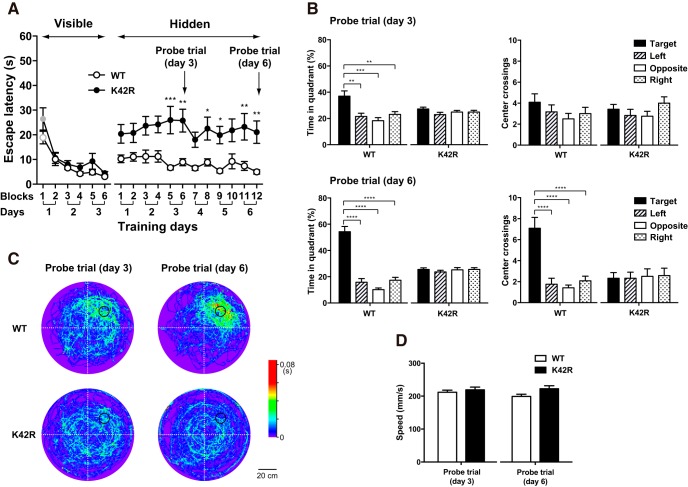
Intact visually guided memory and impaired spatial memory in the Morris water maze tasks in the kinase-dead CaMKIIα (K42R)-KI mouse. ***A***, Escape latencies in the visible platform trials, followed by the hidden platform trials in the water maze tasks in the kinase-dead CaMKIIα (K42R)-KI mouse as compared to the wild-type (WT) mouse. In the visible platform trials (left), both genotypes steadily decreased escape latencies and there was no significant difference between the genotypes. Both genotypes showed significantly shorter latency in the second and later blocks of trials than in the first block (labeled with gray vs black colors, *p* < 0.05, Bonferroni’s *post hoc* test). In the following hidden platform trials (right), latency differed significantly between the genotypes: CaMKIIα (K42R)-KI mice took longer time to reach the platform than wild-type mice did; *p < 0.05, **p < 0.01, ***p < 0.001 between the genotypes, Bonferroni’s *post hoc* test. Open circle, wild-type mice, *n* = 12; closed circle, CaMKIIα (K42R)-KI mice, *n* = 12. Note that the platform location was fixed for each mouse throughout the course of training. ***B***, Probe trials performed on days 3 and 6 after training trials of the day (after 6 and 12 blocks of trials, respectively). Percentage time in each quadrant (left) and the number of center crossings of hypothetical platform locations (right) are shown; **p < 0.01, ***p < 0.001, ****p < 0.0001, Tukey’s *post hoc* test. Closed column, target quadrant; hatched column, left quadrant; open column, opposite quadrant; dotted column, right quadrant. ***C***, Averaged swimming traces of each genotype for 60 s in the probe trials performed on days 3 and 6. The color indicates the average time spent at a certain location (1 × 1 cm square) per animal. The platform location was aligned in the right upper quadrant by rotation and indicated by a black-lined circle. ***D***, Swimming speed measured for 60 s in the probe trials performed on days 3 and 6. There was no significant difference between the genotypes. Open column, wild-type mice; closed column, CaMKIIα (K42R)-KI mice.

In water maze training, mice were individually subjected to two blocks (30–60 min apart) of four trials (30-s intertrial interval) per day with spatial cues. Platform location was fixed for the same group of mice trained on the same days, but altered among different groups of mice (seven to nine mice per group). Mice were gently released into the pool with the starting position changed in each trial, and given 60 s to reach the platform. After climbing onto the platform, mice were allowed to remain there for 30 s. If a mouse was unable to locate the platform within 60 s, the trial was concluded, and the mouse was gently guided onto the platform and remained there for 30 s. After the training, mice were returned to their home cage. The escape latency was calculated as the average time of the four trials per block to reach the platform (Fig. 3*A*).

Mice were subjected to visible platform training for the first 3 d. Subsequently, the visible cue for the platform was removed with the platform location kept intact. The mice were then given hidden platform training for another 6 d. On days 3 and 6 of hidden platform training, the platform was removed after training trials of the day, and a probe trial was performed to assess spatial memory. Mice were released into the pool from the opposite side of the original platform location and allowed to search for the platform location for 60 s, while their swimming paths were recorded. Mice were then gently guided to the original platform location and allowed to sit on the experimenter’s hand for 30 s. The following parameters were measured for each probe trial: (1) the percentage of time spent in each imaginary quadrant of the pool (target, left, opposite and right quadrants; Fig. 3*B*, left); (2) the number of center crossings of the hypothetical platform location in each quadrant (Fig. 3*B*, right); and (3) swimming speed (Fig. 3*D*). Swimming traces were color-coded according to the time spent at a certain location (1 × 1 cm square), and averaged within each genotype after aligning the location of the platform by rotation using Igor Pro 6 software (WaveMetrics; RRID: SCR_000325; Fig. 3*C*).

The escape latencies for visible and hidden platform trainings were compared between the genotypes by using two-way repeated measures ANOVA, followed by Bonferroni’s *post hoc* test where appropriate, *n* = 12 for each genotype (Fig. 3*A*). The percentage of time spent in each imaginary quadrant of the pool and the number of center crossings of the hypothetical platform location in each quadrant in probe trials were analyzed in each genotype by using one-way ANOVA, followed by Tukey’s *post hoc* test where appropriate (Fig. 3*B*). The swimming speed in probe trials was compared between the genotypes by using unpaired *t* test, two-tailed (Fig. 3*D*).

### Fear conditioning

The assessment of electrical footshock sensitivity and fear conditioning were performed in the same clear acrylic chamber (W 300 × D 250 × H 215 mm) equipped with a stainless steel grid floor (3-mm diameter rods, 7 mm apart; O’HARA & CO.). The auditory signal was supplied from a loudspeaker placed on top of the lid of the chamber, and footshock and tone delivery were controlled by using LabVIEW software (National Instruments; RRID: SCR_014325). We employed background contextual fear conditioning using a tone as a conditional stimulus (CS) paired with an unconditional stimulus (US) of a footshock. Background conditioning involves the hippocampus more strongly than conditioning without a tone (Phillips and LeDoux, 1994), and by using this method, we can compare hippocampus- versus amygdala-dependent memory at the same time.

To assess electrical footshock sensitivity, naive mice were individually placed in the chamber, and a series of electrical stimuli was delivered from the grid floor for 2 s (Kojima et al., 2008). The current intensity started from 0.01 mA and gradually increased with one-min interval. The behavioral responses to the electrical stimuli were monitored, and the threshold intensity for a paw flick and/or a step back as a measure of pain sensation and that for vocalization, jump and/or running as a measure of aversive response were determined. The threshold intensities were compared between the genotypes by using unpaired *t* test, two-tailed, *n* = 14 for the wild-type mouse, *n* = 16 for the CaMKIIα (K42R)-KI mouse (Table 2).

**Table 2.** Sensitivity to an electrical footshock in the kinase-dead CaMKIIα (K42R)-KI mouse as compared to the wild-type mouse

	WT	CaMKIIα (K42R)-KI
Step/paw	0.032 ± 0.002	0.031 ± 0.001
Vocal/run/jump	0.061 ± 0.003^a^	0.078 ± 0.003^a^

Threshold currents are expressed in mA.

Step/paw, stepping back and/or paw flick, reflecting pain sensation; Vocal/run/jump, vocalization, running and/or jumping, reflecting aversive reactions.

Significantly different between wild-type (WT) and CaMKIIα (K42R)-KI mice;

a *p* = 0.0001 (unpaired *t* test); WT, *n* = 14; K42R, *n* = 16.

Fear conditioning was conducted by placing naive mice individually in the chamber, while other mice waited in another nearby room where the auditory signal could not be heard. The fear conditioning procedure followed basically as described by Irvine et al. (2005). After an initial exploratory period of 120 s, a tone (70 dB, white noise) was presented for 30 s as a CS, which coterminated with an electrical footshock (0.3 or 0.7 mA, 2 s) as a US (1CS-US or 1CS-US-strong). After 30 s, the mouse was returned to its home cage. The chamber was cleared with 75% ethanol before each session. Under more intense training conditions, mice received three or five CS-US pairings at 60-s intervals (0.3-mA footshock; 3CS-US or 5CS-US). A control experiment consisting of the presentation of a tone alone without a footshock for three times at 60-s intervals (3CS alone) was also conducted to assess fear response to the tone alone without conditioning.

Twenty-four hours after conditioning, the mice were individually reexposed to the conditioning chamber without the auditory signal for 300 s to test for contextual fear memory. Freezing time in the first 180 s was measured (Context). Another 24 h later, the mice were individually placed in a novel context consisting of a metal box (W 235 × D 235 × H 145 mm) installed inside the contextual chamber to assess cued fear memory. Following 180 s without a tone, the tone (70 dB, white noise) was pesented for 180 s. Freezing time in the first 180 s (Cued, Pre-tone) and the last 180 s (Cued, Tone) was measured. Four weeks later, the mice were retested to assess the stability of fear memory (Context; Cued, Pre-tone and Tone).

**Figure 4.**
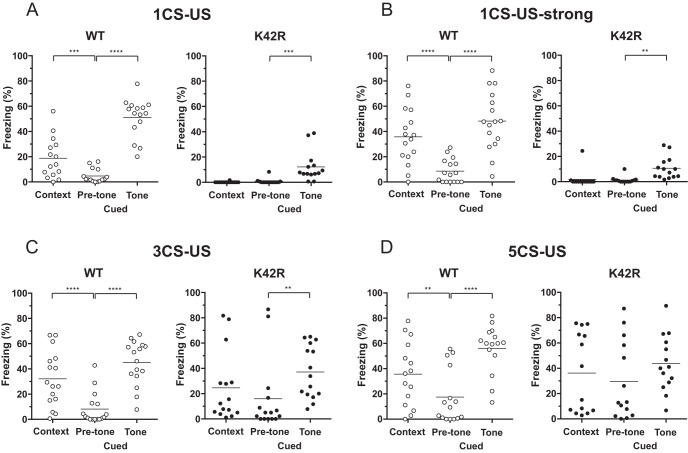
No context discrimination, whereas partially preserved cued fear memory after fear conditioning in the kinase-dead CaMKIIα (K42R)-KI mouse. ***A***, Fear conditioned memory after a single-stimulation protocol with a 30-s tone that coterminated with a 0.3-mA footshock (1CS-US). Contextual fear memory (Context) was tested in the same conditioning chamber 24 h after conditioning. Cued fear memory (Cued) was tested in a different context without (Pre-tone) or with tone (Tone) 48 h after conditioning. Ordinate indicates percentage freezing time for the period of 180 s, and the line in each column expresses the mean. Left, Wild-type (WT) mice showed significantly longer freezing time in the contextual chamber than in the cued chamber without tone, demonstrating context-dependent fear memory. In addition, they showed significantly longer freezing time in the cued chamber with tone than without tone, demonstrating tone-dependent fear memory; *n* = 15. Right, CaMKIIα (K42R)-KI mice showed virtually no freezing in the contextual chamber, as well as in the cued chamber without tone, and there was no significant difference in freezing time between the two conditions. On the other hand, they showed rather small, but significant freezing in the cued chamber with tone, indicating that cued fear memory was formed at least to a certain extent; *n* = 14. ***B***, Fear conditioned memory after a stronger single-stimulation protocol with a 0.7-mA footshock (1CS-US-strong). Basically, similar results were observed as in ***A***. WT, *n* = 16 (left). K42R, *n* = 14 (right). ***C***, Fear conditioned memory after a repeated-stimulation protocol with three pairings of a tone and a 0.3-mA footshock (3CS-US). Left, Wild-type mice showed both context-dependent and tone-dependent freezing; *n* = 16. Right, CaMKIIα (K42R)-KI mice showed increased freezing in all of the three conditions tested, but they still did not show context-dependent freezing, whereas showed tone-dependent freezing, revealing context discrimination deficits; *n* = 15. ***D***, Fear conditioned memory after a repeated stimulation protocol with five pairings of a tone and a 0.3-mA footshock (5CS-US). Left, Wild-type mice again showed both context-dependent and tone-dependent freezing; *n* = 15. Right, CaMKIIα (K42R)-KI mice showed generally increased freezing, and there was no significant difference in percentage freezing time between the three conditions tested, revealing severe context discrimination deficits, leading to generalized fear; *n* = 14; **p < 0.01, ***p < 0.001, ****p < 0.0001, Bonferroni’s *post hoc* test.

**Figure 5.**
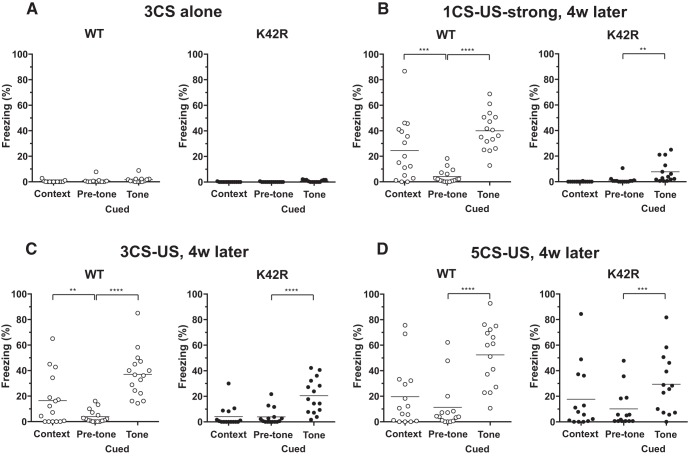
No fear response to tone alone without tone-footshock association, and long-lasting cued fear memory in the kinase-dead CaMKIIα (K42R)-KI mouse. ***A***, Fear response after repeated exposure to tone alone for three times without footshocks (3CS alone). Both wild-type (WT; left) and CaMKIIα (K42R)-KI mice (right) showed almost no freezing in the three conditions tested, demonstrating the specificity of fear response to tone-footshock association in both genotypes (Fig. 4). WT, *n* = 13. K42R, *n* = 13. ***B***, Long-term memory examined four weeks after strong 1CS-US conditioning. The mice in Figure 4*B* were retested four weeks later. Left, Wild-type mice still showed context-dependent and tone-dependent freezing, demonstrating the acquired fear memory was long-lasting; *n* = 16. Right, CaMKIIα (K42R)-KI mice also retained tone-dependent freezing, demonstrating the acquired fear memory was long-lasting; *n* = 14. ***C***, Long-term memory examined four weeks after 3CS-US conditioning. The mice in Figure 4*C* were retested four weeks later. Here again, both wild-type (left) and CaMKIIα (K42R)-KI mice (right) retained once acquired fear memory. WT, *n* = 16. K42R, *n* = 15. ***D***, Long-term memory examined four weeks after 5CS-US conditioning. The mice in Figure 4*D* were retested four weeks later. Left, Wild-type mice still showed tone-dependent freezing, but not context-dependent freezing; *n* = 15. Right, CaMKIIα (K42R)-KI mice revealed tone-dependent freezing this time, indicating that cued fear memory had been acquired from the beginning after 5CS-US conditioning, but had been masked due to generalized fear when tested soon after conditioning (Fig. 4*D*, right). However, when generally increased fear subsided in four weeks, tone-dependent freezing became apparent; *n* = 14; **p < 0.01, ***p < 0.001, ****p < 0.0001, Bonferroni’s *post hoc* test.

Freezing was defined as complete immobility other than respiration and used as an index of fear (Frankland et al., 1998). Freezing time was measured manually with the experimenter blind to the genotype and expressed as a percentage of time spent freezing during the specified period of 180 s. Values for percentage freezing time were compared between the three conditions tested (Context; Cued, Pre-tone and Tone) in each genotype by using one-way repeated measures ANOVA, followed by Bonferroni’s *post hoc* test where appropriate. Through this comparison, we could accurately evaluate hippocampus-dependent context discrimination and amygdala-dependent cued fear memory at the same time. Context discrimination is a more sensitive measure of hippocampal dysfunction as reported by Frankland et al. (1998). The number of animals used were: the wild-type mouse, *n* = 15, 16, 16, 15, and 13 for 1CS-US, 1CS-US-strong, 3CS-US, 5CS-US, and 3CS alone, respectively; the CaMKIIα (K42R)-KI mouse, *n* = 14, 14, 15, 14, and 13 for 1CS-US, 1CS-US-strong, 3CS-US, 5CS-US, and 3CS alone, respectively (Figs. 4, 5). Tone-dependent freezing (Cued, Tone) was compared between the genotypes by using unpaired *t* test, two-tailed where appropriate. The behavior of the mice during fear conditioning and tests was also videotaped.

### Experimental design and statistical analyses

Details of the experimental design and statistics are described in the above sections. Statistical analysis was performed by using GraphPad Prism 6 software (GraphPad Software; RRID: SCR_002798). Statistical significance was set at *p* < 0.05. Data are expressed as a mean ± SEM or as a scatter plot with a mean.

## Results

### Kinase activity and protein levels of CaMKII were indistinguishable between the hippocampus and amygdala in the wild-type mouse and in the kinase-dead CaMKIIα (K42R)-KI mouse

We first compared the kinase activity and protein levels of CaMKII between the hippocampus and amygdala in the wild-type mouse and in the kinase-dead homozygous CaMKIIα (K42R)-KI mouse (Figs. 1, 2; Table 1). As for CaMKII activity, no significant difference was observed between the hippocampus and amygdala in the wild-type mouse (total activity, *t*_(10)_ = 0.7433, *p* = 0.4744; autonomous activity, *t*_(10)_ = 0.5082, *p* = 0.6223; unpaired *t* test, *n* = 6; Fig. 1, left), indicating that the total amounts of CaMKII composed of CaMKIIα and CaMKIIβ, the two major isoforms in the brain, are indistinguishable between the two brain regions. In the same way, no significant difference was observed between the two brain regions in the CaMKIIα (K42R)-KI mouse (total activity, *t*_(10)_ = 1.812, *p* = 0.1001; autonomous activity, *t*_(10)_ = 0.8094, *p* = 0.4371; unpaired *t* test, *n* = 6; Fig. 1, right), demonstrating that the remaining CaMKII activity derived from intact CaMKIIβ is indistinguishable between the two brain regions. This also indicates that the amount of CaMKIIβ is basically the same between the two brain regions in the CaMKIIα (K42R)-KI mouse.

We then compared CaMKIIα and CaMKIIβ protein levels between the genotypes in the hippocampus and in the amygdala by quantitative immunoblot analyses (Fig. 2; Table 1). The CaMKIIβ protein level in the CaMKIIα (K42R)-KI mouse was unchanged from that in the wild-type mouse in the both brain regions (hippocampus, *t*_(5)_ = 0.2667, *p* = 0.8004; amygdala, *t*_(5)_ = 0.8194, *p* = 0.4498; one sample *t* test, *n* = 6; Table 1). Combined with activity data, the amount of CaMKIIβ, and also of CaMKIIα, in the wild-type mouse is considered to be the same between the two brain regions. In addition, there seem to be no compensatory changes in the CaMKIIβ level in the CaMKIIα (K42R)-KI mouse in either brain regions.

It should be noted that the basic characteristics of CaMKII observed in the hippocampus and the amygdala of the kinase-dead CaMKIIα (K42R)-KI mouse, i.e., a profound decrease in CaMKII activity (Fig. 1), a moderate decrease in the phospho-T286-CaMKIIα level and a slight decrease in the CaMKIIα protein level (Fig. 2; Table 1), compared to those in the wild-type mouse, are all in accordance with our previous observations in the forebrain of this KI mouse (Yamagata et al., 2009).

### Impaired spatial memory and intact visually guided memory in the Morris water maze tasks in the kinase-dead CaMKIIα (K42R)-KI mouse

To examine whether hippocampus-dependent spatial memory is indeed impaired in the CaMKIIα (K42R)-KI mouse, we first performed the Morris water maze tasks (Fig. 3). In the visible platform trials, both wild-type and CaMKIIα (K42R)-KI mice showed a steady shortening of escape latencies through the course of training trials, and there was no significant difference between the genotypes (interaction, *F*_(5,110)_ = 0.8647, *p* = 0.5074; genotype, *F*_(1,22)_ = 3.226, *p* = 0.0862; blocks of trials, *F*_(5,110)_ = 24.00, *p* < 0.0001; *n* = 12 for each genotype; two-way repeated measures ANOVA; Fig. 3*A*, left), indicating that visual and motor abilities and motivation for escape are all intact in the CaMKIIα (K42R)-KI mouse. In fact, both genotypes took <11 s on average to reach the visible platform in the second and later blocks of trials, which was significantly shorter than the latency in the first block of trials (wild type: 1st vs 2nd, *p* = 0.01; 1st vs 3rd, *p* = 0.0001; 1st vs 4th, 5th, and 6th, *p* < 0.0001; K42R: 1st vs 2nd, 3rd, 4th, 5th, and 6th, *p* < 0.0001; Bonferroni’s *post hoc* test).

Next, in the following hidden platform trials with the platform location kept intact, the difference between the genotypes became obvious (interaction, *F*_(11,242)_ = 0.7418, *p* = 0.6977; genotype, *F*_(1,22)_ = 16.53, *p* = 0.0005; blocks of trials, *F*_(11,242)_ = 1.254, *p* = 0.2522; two-way repeated measures ANOVA; Fig. 3*A*, right). Wild-type mice took <12 s on average to reach the platform throughout the trials, suggesting that they still remembered the platform location, whereas CaMKIIα (K42R)-KI mice took >18 s on average to reach the platform. *Post hoc* test revealed a significant difference in the latency between the genotypes in the 5th, 6th, 8th, 9th, 11th, and 12th blocks of trials (*p* = 0.0005, 0.0041, 0.0415, 0.0235, 0.0087, and 0.0072, respectively, Bonferroni’s *post hoc* test).

In addition, probe trials performed on days 3 and 6 after training trials of the day revealed that wild-type mice spent significantly longer time in the target quadrant than in other quadrants (day 3, *F*_(3,44)_ = 8.641, *p* = 0.0001, one-way ANOVA; target vs left, *p* = 0.0018; target vs opposite, *p* = 0.0001; target vs right, *p* = 0.0057; Tukey’s *post hoc* test; day 6, *F*_(3,44)_ = 53.15, *p* < 0.0001, one-way ANOVA; target vs left, opposite and right, *p* < 0.0001; Tukey’s *post hoc* test; Fig. 3*B*, left), demonstrating that spatial memory was formed in wild-type mice. On the other hand, CaMKIIα (K42R)-KI mice spent equal time in each quadrant (day 3, *F*_(3,44)_ = 1.434, *p* = 0.2457; day 6, *F*_(3,44)_ = 0.4409, *p* = 0.7249; one-way ANOVA; Fig. 3*B*, left), demonstrating that no spatial memory was formed in CaMKIIα (K42R)-KI mice. Furthermore, in wild-type mice, the number of center crossings of the hypothetical platform location was significantly higher in the target quadrant than in other quadrants in the probe trial performed on day 6 (*F*_(3,44)_ = 17.17, *p* < 0.0001, one-way ANOVA; target vs left, opposite and right, *p* < 0.0001; Tukey’s *post hoc* test), but not on day 3 (*F*_(3,44)_ = 1.005, *p* = 0.3997, one-way ANOVA; Fig. 3*B*, right), indicating that wild-type mice steadily acquired more accurate spatial memory of the hidden platform location as the training proceeded. In contrast, CaMKIIα (K42R)-KI mice did not display any preference for locations of center crossings (day 3, *F*_(3,44)_ = 1.183, *p* = 0.3271; day 6, *F*_(3,44)_ = 0.03869, *p* = 0.9897; one-way ANOVA; Fig. 3*B*, right). Averaged color-coded swimming traces of each genotype in the probe trials on days 3 and 6 clearly demonstrate the preference for the target location in wild-type mice and the absence of such preference in CaMKIIα (K42R)-KI mice (Fig. 3*C*). They also show the improvement of performance with further training from day 3 to day 6 in wild-type mice, but not in CaMKIIα (K42R)-KI mice.

Finally, swimming speed measured in probe trials performed on days 3 and 6 was similar between the genotypes (day 3, *t*_(22)_ = 0.7064, *p* = 0.4874; day 6, *t*_(22)_ = 2.068, *p* = 0.0506; unpaired *t* test), indicating that swimming performance itself was normal in the CaMKIIα (K42R)-KI mouse (Fig. 3*D*). All these results clearly demonstrate that the CaMKIIα (K42R)-KI mouse was severely and specifically impaired in hippocampus-dependent spatial memory, whereas their visually guided memory was kept intact.

### Severely impaired context discrimination, whereas impaired but partially preserved cued fear memory in the kinase-dead CaMKIIα (K42R)-KI mouse

We next examined fear-conditioned memory to compare hippocampus- and amygdala-dependent memory in the CaMKIIα (K42R)-KI mouse. To do that, we first determined the sensitivity to an electrical footshock in the CaMKIIα (K42R)-KI mouse as compared to that in the wild-type mouse (Table 2). The threshold currents for stepping back and/or paw flick, which are the behavioral signs of pain sensation, were indistinguishable between the genotypes (*t*_(28)_ = 0.6530, *p* = 0.5191; unpaired *t* test, *n* = 14 for wild-type mice, *n* = 16 for KI mice). On the other hand, the threshold currents for vocalization, running and/or jumping, which represent aversive reactions to a footshock, was slightly higher in CaMKIIα (K42R)-KI mice than in wild-type mice (*t*_(28)_ = 4.436, *p* = 0.0001; unpaired *t* test). To overcome the difference, we employed the stimulation intensity of 0.3 mA or higher that was far above the threshold for aversive sensation for both genotypes in fear conditioning. It should be noted that in conditioning, no freezing was observed during the first two minutes before the tone in either genotypes.

We first tried single stimulation protocol, i.e., one paring of CS and US (1CS-US) consisting of a tone lasting for 30 s that coterminated with a footshock of 0.3 mA for 2 s (Fig. 4*A*). When tested 24 h (Context) and 48 h (Cued) after conditioning, wild-type mice showed significantly longer freezing time in the contextual chamber (Context in Fig. 4*A*, left), as well as in the cued chamber with tone (Cued, Tone), than in the cued chamber without tone (Cued, Pre-tone; *F*_(2,28)_ = 89.16, *p* < 0.0001, *n* = 15, one-way repeated measures ANOVA; context vs pre-tone, *p* = 0.0009; pre-tone vs tone, *p* < 0.0001; Bonferroni’s *post hoc* test; Fig. 4*A*, left), demonstrating the formation of both context-dependent and tone-dependent fear memory in the wild-type mouse. On the other hand, CaMKIIα (K42R)-KI mice showed almost no freezing in the contextual chamber (Context in Fig. 4*A*, right) and in the cued chamber without tone (Cued, Pre-tone), whereas they showed rather small but significant freezing in the cued chamber with tone (Cued, Tone; *F*_(2,26)_ = 15.65, *p* < 0.0001, *n* = 14, one-way repeated measures ANOVA; context vs pre-tone, *p* > 0.9999; pre-tone vs tone, *p* = 0.0001; Bonferroni’s *post hoc* test; Fig. 4*A*, right), demonstrating that context-dependent fear memory was not formed, but tone-dependent fear memory was formed to a certain extent, although it was significantly less than that observed in wild-type mice (Cued, Tone; *t*_(27)_ = 7.566, *p* < 0.0001; unpaired *t* test).

We then tried stronger 1CS-US conditioning using a footshock of 0.7 mA (1CS-US-strong) in another groups of mice to examine whether stronger stimulation protocol could compensate for memory deficits in the CaMKIIα (K42R)-KI mouse (Fig. 4*B*). After such stronger conditioning, however, the results were basically the same: Wild-type mice showed both context-dependent and tone-dependent freezing (*F*_(2,30)_ = 46.95, *p* < 0.0001, *n* = 16, one-way repeated measures ANOVA; context vs pre-tone, *p* < 0.0001; pre-tone vs tone, *p* < 0.0001; Bonferroni’s *post hoc* test; Fig. 4*B*, left), whereas CaMKIIα (K42R)-KI mice did not show context-dependent freezing, but showed tone-dependent freezing to a certain extent (*F*_(2,26)_ = 9.601, *p* = 0.0008, *n* = 14, one-way repeated measures ANOVA; context vs pre-tone, *p* > 0.9999; pre-tone vs tone, *p* = 0.0012; Bonferroni’s *post hoc* test; Fig. 4*B*, right), the latter of which was still significantly less than that observed in wild-type mice (Cued, Tone; *t*_(28)_ = 5.687, *p* < 0.0001; unpaired *t* test). Thus, stronger conditioning did not compensate for memory deficits in the CaMKIIα (K42R)-KI mouse.

We next performed repeated stimulation protocols consisting of three or five parings of CS-US (3CS-US or 5CS-US), using a footshock of 0.3 mA in another groups of mice to examine whether repeated stimulation could compensate for memory deficits in the CaMKIIα (K42R)-KI mouse (Fig. 4*C*,*D*). After 3CS-US conditioning, wild-type mice again showed context-dependent and tone-dependent freezing (*F*_(2,30)_ = 37.41, *p* < 0.0001, *n* = 16, one-way repeated measures ANOVA; context vs pre-tone, *p* < 0.0001; pre-tone vs tone, *p* < 0.0001; Bonferroni’s *post hoc* test; Fig. 4*C*, left). CaMKIIα (K42R)-KI mice, this time, showed increased freezing in all of the three conditions tested, but still did not show context-dependent freezing, whereas showed tone-dependent freezing (*F*_(2,28)_ = 5.230, *p* = 0.0117, *n* = 15, one-way repeated measures ANOVA; context vs pre-tone, *p* = 0.3989; pre-tone vs tone, *p* = 0.0065; Bonferroni’s *post hoc* test; Fig. 4*C*, right), revealing context discrimination deficits. The extent of their tone-dependent freezing was comparable to that observed in wild-type mice (Cued, Tone; *t*_(29)_ = 1.143, *p* = 0.2624; unpaired *t* test). Next, after 5CS-US conditioning, wild-type mice still showed context-dependent and tone-dependent freezing (*F*_(2,28)_ = 23.13, *p* < 0.0001, *n* = 15, one-way repeated measures ANOVA; context vs pre-tone, *p* = 0.0067; pre-tone vs tone, *p* < 0.0001; Bonferroni’s *post hoc* test; Fig. 4*D*, left). CaMKIIα (K42R)-KI mice, on the other hand, showed generally increased freezing, and this time, freezing was neither context-dependent nor tone-dependent (*F*_(2,26)_ = 2.655, *p* = 0.0893, *n* = 14, one-way repeated measures ANOVA; Fig. 4*D*, right). Thus, repeated stimulation protocols did not compensate for memory deficits, but instead revealed severe context discrimination deficits, leading to generalized fear, in the CaMKIIα (K42R)-KI mouse.

To rule out the possibility that the CaMKIIα (K42R)-KI mouse may display tone-dependent freezing without tone-footshock association, we exposed another groups of mice to tone alone for three times without footshocks (3CS alone) and tested their freezing response just as in the case after fear conditioning (Fig. 5*A*). Virtually no freezing was observed in CaMKIIα (K42R)-KI mice (*n* = 13; Fig. 5*A*, right), as well as in wild-type mice (*n* = 13; Fig. 5*A*, left), in all of the three conditions tested (percentage freezing time, <2% on average in any cases). Thus, tone-dependent fear responses in both genotypes were indeed derived from tone-footshock association, and not from tone alone.

So far, the results demonstrate that the CaMKIIα (K42R)-KI mouse could form cued fear memory at least to a certain extent, whereas they could not form contextual fear memory or discriminate context difference irrespective of stimulus intensity and of repetition of a tone-footshock paring in the conditioning.

### Cued fear memory once acquired was long-lasting in the kinase-dead CaMKIIα (K42R)-KI mouse

Does the cued fear memory once formed in the CaMKIIα (K42R)-KI mouse last long? To solve that question, we retested fear-conditioned mice four weeks later (Fig. 5*B–D*). Four weeks after strong 1CS-US conditioning, wild-type mice still displayed context-dependent and tone-dependent freezing (*F*_(2,30)_ = 32.04, *p* < 0.0001, *n* = 16, one-way repeated measures ANOVA; context vs pre-tone, *p* = 0.0002; pre-tone vs tone, *p* < 0.0001; Bonferroni’s *post hoc* test; Fig. 5*B*, left). In addition, CaMKIIα (K42R)-KI mice still preserved small but significant tone-dependent freezing (*F*_(2,26)_ = 9.253, *p* = 0.0009, *n* = 14, one-way repeated measures ANOVA; context vs pre-tone, *p* > 0.9999; pre-tone vs tone, *p* = 0.0045; Bonferroni’s *post hoc* test; Fig. 5*B*, right), the extent of which was significantly less than that observed in wild-type mice (Cued, Tone; *t*_(28)_ = 7.115, *p* < 0.0001; unpaired *t* test). Thus, in both genotypes, memory once formed lasted long.

Next, four weeks after 3CS-US conditioning, wild-type mice again displayed context-dependent and tone-dependent freezing (*F*_(2,30)_ = 39.68, *p* < 0.0001, *n* = 16, one-way repeated measures ANOVA; context vs pre-tone, *p* = 0.0044; pre-tone vs tone, *p* < 0.0001; Bonferroni’s *post hoc* test; Fig. 5*C*, left). CaMKIIα (K42R)-KI mice, on the other hand, showed considerably decreased freezing in the three conditions tested, but still displayed tone-dependent freezing without context-dependent freezing (*F*_(2,28)_ = 15.25, *p* < 0.0001, *n* = 15, one-way repeated measures ANOVA; context vs pre-tone, *p* > 0.9999; pre-tone vs tone, *p* < 0.0001; Bonferroni’s *post hoc* test; Fig. 5*C*, right). The extent of tone-dependent freezing was significantly less than that observed in wild-type mice (Cued, Tone; *t*_(29)_ = 2.872, *p* = 0.0076; unpaired *t* test). Here again, fear conditioned memory once formed was long-lasting in both genotypes.

Finally, four weeks after 5CS-US conditioning, wild-type mice still preserved tone-dependent freezing, but not context-dependent freezing any more (*F*_(2,28)_ = 32.57, *p* < 0.0001, *n* = 15, one-way repeated measures ANOVA; context vs pre-tone, *p* = 0.2710; pre-tone vs tone, *p* < 0.0001; Bonferroni’s *post hoc* test; Fig. 5*D*, left), indicating that context discrimination is more fragile than cued fear memory in the long-term in the wild-type mouse. CaMKIIα (K42R)-KI mice, on the other hand, still showed rather increased levels of freezing in the three conditions tested, while this time, they did display tone-dependent freezing, which had not been observed 48 h after conditioning, but without context-dependent freezing (*F*_(2,26)_ = 10.95, *p* = 0.0004, *n* = 14, one-way repeated measures ANOVA; context vs pre-tone, *p* = 0.1603; pre-tone vs tone, *p* = 0.0002; Bonferroni’s *post hoc* test; Fig. 5*D*, right). The extent of tone-dependent freezing was again less than that observed in wild-type mice (Cued, Tone; *t*_(27)_ = 2.583, *p* = 0.0155; unpaired *t* test). The results indicate that the CaMKIIα (K42R)-KI mouse had formed cued fear memory from the beginning after 5CS-US conditioning, but its manifestation had been masked by generally increased fear when tested soon after conditioning. However, when generalized fear subsided in four weeks, cued fear memory became apparent.

All these results clearly demonstrate that cued fear memory once acquired was long-lasting, not only in the wild-type mouse, but also in the CaMKIIα (K42R)-KI mouse.

## Discussion

**Figure 6.**
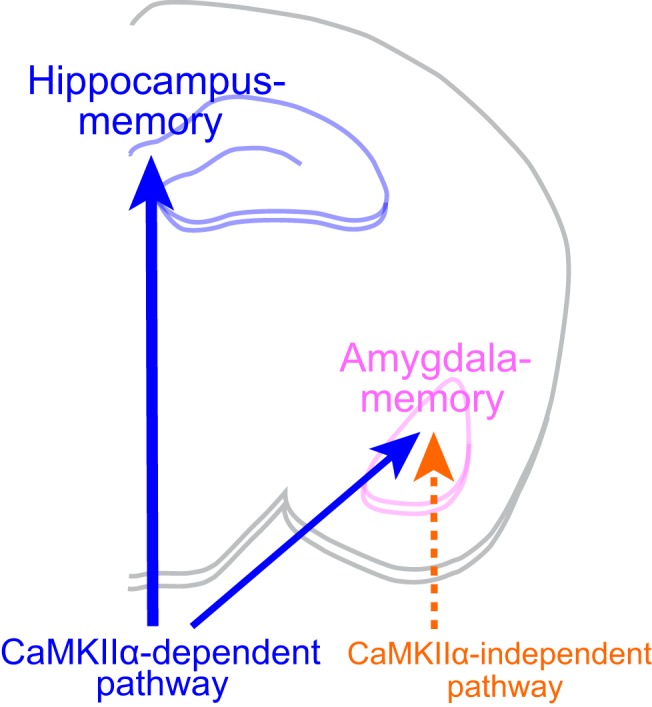
Schematic illustration showing differential involvement of kinase activity of CaMKIIα in hippocampus- and amygdala-dependent memory. Hippocampus-dependent memory is mediated exclusively by the CaMKIIα activity-dependent pathway, whereas amygdala-dependent memory is mediated not only by the CaMKIIα activity-dependent pathway, but also by a CaMKIIα activity-independent pathway, the candidate of which may include CaMKIIβ activity or *de novo* synaptogenesis, such as generation of multi-innervated dendritic spines (see text in detail).

In this study, we have shown that the kinase-dead homozygous CaMKIIα (K42R)-KI mouse retained intact visually guided memory, but was severely impaired in hippocampus-dependent spatial memory in the Morris water maze tasks (Fig. 3). In addition, the CaMKIIα (K42R)-KI mouse was severely impaired in hippocampus-dependent context discrimination in fear memory, whereas amygdala-dependent cued fear memory was impaired but preserved to some extent, and once formed cued fear memory lasted as long as four weeks (Figs. 4, 5). These results indicate that hippocampus-dependent memory was severely impaired, but amygdala-dependent memory was partially spared in the kinase-dead CaMKIIα (K42R)-KI mouse. Such difference was not due to differential expression of CaMKIIα between the hippocampus and the amygdala because biochemical analyses revealed that both kinase activity and protein levels of CaMKII were indistinguishable between the two brain regions (Figs. 1, 2; Table 1). All these results indicate that the involvement of kinase activity of CaMKIIα in the mechanisms of learning and memory seems to be somewhat different between the types of memory: It is indispensable for hippocampus-dependent memory, whereas although important, not necessarily indispensable for amygdala-dependent memory (Fig. 6).

The requirement of CaMKIIα in the acquisition of hippocampus-dependent memory has been shown by using various kinds of CaMKIIα KO and KI mice (Silva et al., 1992; Giese et al., 1998; Elgersma et al., 2002; Yamagata et al., 2009; Achterberg, et al., 2014). In addition to the homozygous CaMKIIα KO mouse (Silva et al., 1992; Elgersma et al., 2002), the autophosphorylation-deficient CaMKIIα (T286A)-KI mouse that lacks persistent activation of CaMKIIα (Giese et al., 1998; Irvine et al., 2005), and the autophosphorylation-mimicking CaMKIIα (T305D)-KI mouse that lacks both kinase activity and postsynaptic association of CaMKIIα (Elgersma et al., 2002) showed profound impairments in spatial memory in the Morris water maze tasks and in contextual fear memory after a single-footshock conditioning. Besides, our kinase-dead CaMKIIα (K42R)-KI mouse that lacks kinase activity but retains activity-dependent postsynaptic translocational ability of CaMKIIα was severely impaired in inhibitory avoidance memory, a form of memory dependent on the hippocampus (Yamagata et al., 2009). A recent study using the region-specific and time-specific conditional CaMKIIα KO mice also showed the necessity of CaMKIIα protein in the forebrain and at the time of learning for the acquisition of spatial and contextual memory (Achterberg, et al., 2014). All these results showed that proper functioning of CaMKIIα is essential for hippocampus-dependent memory to be formed, which is in accordance with our present study.

On the other hand, the CaMKIIα (T286A)-KI mouse could acquire wild-type levels of contextual and cued fear memory when multiple footshocks were delivered during conditioning (Irvine et al., 2005, 2011). This is a clear contrast to our current results showing that repetitive training was ineffective to overcome memory deficits, but revealed context discrimination deficits and caused generalized fear response in the kinase-dead CaMKIIα (K42R)-KI mouse (Fig. 4*C*,*D*). Besides, when retested four weeks later, generalized fear subsided considerably, and cued fear memory, but not context discrimination, was observed in the kinase-dead CaMKIIα (K42R)-KI mouse (Fig. 5*D*). The differential effects of repeated training observed between the CaMKIIα (K42R)-KI mouse and the CaMKIIα (T286A)-KI mouse could be explained by biochemical differences between the two mutations or by a dominant-negative effect of kinase-dead mutation. From the biochemical point of view, K42R mutation completely inactivates the kinase, whereas T286A mutation dose not, i.e., CaMKIIα (T286A) can be fully activated in the presence of Ca^2+^/calmodulin, but once Ca^2+^/calmodulin is detached from it, it completely loses its activity. Repetitive stimulation in repeated training given to the CaMKIIα (T286A)-KI mouse may induce enough kinase activity for an extended period of time, which seems to be necessary for the acquisition of memory. A recent report showing that increasing the stimulation frequency could prolong the activation state of CaMKIIα (T286A) and enabled the induction of LTP at the synaptic level supports this notion (Chang et al., 2017). On the other hand, another kinase-dead mutant, CaMKIIα (K42M) was shown to have a dominant-negative effect in the control of synaptic strength when transfected into organotypic hippocampal slice cultures (Kabakov and Lisman, 2015). Besides, viral injection of CaMKIIα (K42M) into the CA1 region of the hippocampus in rats caused erasure of acquired fear memory in conditioned place avoidance, another hippocampus-dependent memory task in which an animal receives a footshock every time it enters a shock zone (Rossetti et al., 2017). These reports raise a possibility that kinase-dead mutation of CaMKIIα may compromise the role of intact other isoforms of CaMKII, especially that of CaMKIIβ in the same CaMKII holoenzyme, and cause stronger memory deficits than expected. This may be another reason why the kinase-dead CaMKIIα (K42R)-KI mouse in the current study showed severe memory deficits even after repeated training, which was in contrast to the case of the autophosphorylation-deficient CaMKIIα (T286A)-KI mouse.

As for amygdala-dependent memory, several studies implicated a similar involvement of CaMKIIα to the mechanism as in hippocampus-dependent memory (Rodrigues et al., 2004; Irvine et al., 2005). Auditory fear conditioning in rats increased T286-autophosphorylation of CaMKIIα in lateral amygdala spines, and the injection of a CaMKII inhibitor, KN-62 into the amygdala impaired acquisition of both auditory and contextual fear memory (Rodrigues et al., 2004). In addition, as described above, repeated training could compensate for deficits not only in contextual, but also in cued fear memory in the CaMKIIα (T286A)-KI mouse (Irvine et al., 2005, 2011). Thus, CaMKIIα had been thought to play a similar mechanistic role in the acquisition of hippocampus- and amygdala-dependent memory. Such assumption was further supported by a recent study showing that the injection of a photoactivatable CaMKII inhibitory peptide into the mouse amygdala caused deficits in inhibitory avoidance memory tested at least 1 h after training (Murakoshi et al., 2017). However, our present study clearly demonstrated that although small, but distinct amygdala-dependent cued fear memory was observed in the kinase-dead CaMKIIα (K42R)-KI mouse, which was stable and long-lasting. Thus, CaMKIIα activity seems to be differentially involved in the two types of memory: Hippocampus-dependent memory is mediated exclusively by the CaMKIIα activity-dependent pathway, whereas amygdala-dependent memory is mediated not only by the CaMKIIα activity-dependent pathway, but also by a CaMKIIα activity-independent pathway, at least to some extent (Fig. 6). Such differential involvement seems to be reasonable given the fact that amygdala-dependent fear-related memory is essential for the survival of animals, and the existence of a secondary pathway to achieve it is preferable. On the other hand, hippocampus-dependent spatial memory and context discrimination contributes to the efficiency of life, but is not necessarily essential for the survival of animals, and thus, redundant pathway may not be necessary.

Then, what is the secondary, CaMKIIα activity-independent pathway that may be involved in the acquisition of amygdala-dependent fear memory? One possibility could be the involvement of other isoforms of CaMKII, i.e., CaMKIIβ, CaMKIIγ, and CaMKIIδ that are derived from distinct genes. Among them, CaMKIIβ is suited to have a complementary role to CaMKIIα, because CaMKIIβ is the second major CaMKII isoform in the forebrain and shows neuron-specific expression, besides CaMKIIα. Such assumption is indeed supported by the above-mentioned previous studies using CaMKII inhibitors that are effective to all isoforms of CaMKII, including CaMKIIα and CaMKIIβ: Injection of KN-62 and photoactivatable inhibitory peptide into the amygdala showed inhibitory effects on fear conditioned memory and inhibitory avoidance memory, respectively (Rodrigues et al., 2004; Murakoshi et al., 2017). Thus, there may be a synergistic role of CaMKIIα and CaMKIIβ activity in the acquisition of amygdala-dependent memory. It is interesting to note that CaMKIIβ was shown to play a nonenzymatic role in hippocampus-dependent memory, i.e., its kinase activity is not necessary, but its molecular existence itself seems to be required for proper targeting of CaMKIIα to the synapse (Borgesius, et al., 2011). Thus, CaMKIIβ may play an enzymatic role, in addition to a nonenzymatic role, in the acquisition of amygdala-dependent memory.

Another possibility could be *de novo* synaptogenesis, such as generation of multi-innervated dendritic spines, which is analogous to the one described for the formation of hippocampus-dependent contextual fear memory in the CaMKIIα (T286A)-KI mouse after repeated training (Radwanska et al., 2011). In the latter case, multi-trial training induced long-term contextual fear memory that was accompanied by PSD95 up-regulation and synaptogenesis in the CA1 region of the dorsal hippocampus. A similar synaptogenetic changes in the amygdala may account for the formation of long-lasting cued fear memory in the absence of CaMKIIα activity after repeated training, as observed in the kinase-dead CaMKIIα (K42R)-KI mouse in the current study. It is interesting to note that in the above-mentioned study injecting a photoactivatable CaMKII inhibitory peptide into the mouse amygdala, 1-h memory was indeed affected, but 24-h memory tests showed mixed results (Murakoshi et al., 2017), indicating possible existence of another pathway that is unrelated to CaMKII activity. Further studies are necessary to identify the exact molecular mechanisms involved in the acquisition of amygdala-dependent memory, other than those involving CaMKIIα activity (Fig. 6).

One more interesting observation in this study is that the kinase-dead CaMKIIα (K42R)-KI mouse displayed severe context discrimination deficits, leading to generalized fear after repeated CS-US conditioning (Fig. 4*C*,*D*). Why fear response was generalized after intense training in the KI mouse? Two factors may account for this phenomenon. One is severely impaired hippocampal function caused by the lack of CaMKIIα kinase activity. In the CaMKIIα (K42R)-KI mouse, hippocampal LTP is severely impaired (Yamagata et al., 2009), which will hamper the acquisition of accurate spatial memory as shown in this study (Fig. 3). Besides, the hippocampus is known to play a key role in integrating discrete contextual elements processed in different subcortical and cortical regions and in encoding a configured representation of the context in fear conditioning (Holland and Bouton, 1999; Maren et al., 2013). Normally, individual contextual elements are superseded by a representation of the context formed in the hippocampus, but with hippocampal damage, each stimulus elements that make up the context encoded outside the hippocampus seems to be used for conditioning and associated with noxious US within the amygdala. Such multiple elemental associations may occur in the CaMKIIα (K42R)-KI mouse after strong activation of the amygdala by repeated training. The other factor is impaired amygdala function by the lack of CaMKIIα kinase activity. CaMKII activity is known to play an important role in NMDA receptor-dependent LTP at thalamic input synapses to the lateral amygdala (Rodrigues et al., 2004; Johansen et al., 2011). A recent, elegant study showed that auditory fear conditioning is mediated by input-specific LTP (Kim and Cho, 2017), which enables discrimination of dangerous stimuli from safe ones (Maren, 2017), and could apply to contextual conditioning as well. With the lack of CaMKIIα kinase activity, input specificity of LTP and of conditioning would be lost, and under such a situation, strong activation of the amygdala by repeated training could cause potentiation of nearby, unrelated synapses, leading to generalized fear response without discrimination between dangerous and safe stimulus environments. Thus, impaired hippocampal contextual configuration and impaired input-specificity in the amygdala may account for fear generalization in the CaMKIIα (K42R)-KI mouse after intense training. It is interesting to note that hippocampal dysfunction is proposed to be related to generalized fear memory in human patients with posttraumatic stress disorder (PTSD) as well (Acheson et al., 2012).

Increased fear memory generalization is one of the characteristics of PTSD and other anxiety-related disorders (Mahan and Ressler, 2012; Pitman et al., 2012). Generalized fear response after repeated training in the kinase-dead CaMKIIα (K42R)-KI mouse makes us think if this KI mouse can serve as an animal model of PTSD. However, it should be noted that when retested four weeks after repeated CS-US conditioning, the CaMKIIα (K42R)-KI mouse showed considerably reduced fear response, and fear generalization was no longer observed (Fig. 5*D*). Natural recovery from fear generalization without extinction training indicates that extinction of increased fear memory seems to be preserved in the CaMKIIα (K42R)-KI mouse, which does not fit as a PTSD model, because in PTSD, fear generalization is sustained and difficult to be extinguished (Siegmund and Wotjak, 2006; Yehuda and LeDoux, 2007). Rather, generalized fear response observed in the CaMKIIα (K42R)-KI mouse indicates that the lack of kinase activity of CaMKIIα could be one of the predispositions or risk factors toward the development of PTSD. Further investigation of the kinase-dead CaMKIIα (K42R)-KI mouse would lead to the understanding and novel therapeutics of PTSD and other abnormal fear memory-associated disorders.

## Synthesis

Reviewing Editor: Daniela Schiller, Mount Sinai School of Medicine Decisions are customarily a result of the Reviewing Editor and the peer reviewers coming together and discussing their recommendations until a consensus is reached. When revisions are invited, a fact-based synthesis statement explaining their decision and outlining what is needed to prepare a revision will be listed below. The following reviewer(s) agreed to reveal their identity: Karl Giese, Geeske van Woerden. Note: If this manuscript was transferred from JNeurosci and a decision was made to accept the manuscript without peer review, a brief statement to this effect will instead be what is listed below.

The authors studied hippocampus- and amygalda-dependent memory formation in a knockin mutant mouse line that blocks alphaCaMKII kinase activity. Studies with these knockin mutants are very important as the general alphaCaMKII knockout mice suffer from a compensatory translocation of betaCaMKII into the PSD, which does not appear to occur in these knockin mice. The literature on CaMKIIa and the involvement in learning and memory is already substantial. The fact that both hippocampal- as well as amygdala-dependent learning and memory is severely dependent on CaMKIIa-dependent activity is also already known as indicated by the authors themselves when citing Rodrigues et al., 2004 and Murakoshi et al., 2017. In addition, these 2 papers both show residual learning and memory present, which is similar to what the authors here show.

In the current report, the authors found evidence that despite the lack of alphaCaMKII activity a long-lasting tone fear memory can be generated. In contrast, the mutants have substantial impairments in hippocampus-dependent spatial memory formation and contextual discrimination. Further, the mice seem to exhibit increased generalized fear. This leads to the conclusion that, amaygdala-, but not hippocampus-dependent memory can be formed in absence of alphaCaMKII activity and that the kinase is required for establishing specific memories. This finding is important not only for understanding the molecular bases of memory storage, but also for developing treatments of PTSD. The work suggests that massed training can overcome a deficit in tone fear conditioning but not contextual fear conditioning. This is a different phenotype from the T286A mutants, which have normal pre-tone freezing; and other CaMKII mutants were not tested as extensively in fear conditioning. Thus, the result suggests that under strong fear stimulation generalized fear is generated; this is interesting as in PTSD fear is generalized. The authors should discuss this point in greater detail. In fact the authors may wish to quantify the amount of generalized freezing per animal. Is it possible that this enhanced generalized freezing is due to loss of input specificity of synaptic plasticity? It is important to substantiate this point because the mutants appear to be a great model of PTSD.

The amount of freezing behavior in the cued conditioning of the mutant compared to the wt is still significantly reduced in all the fear conditioning experiments performed. For example, in the 1CS-US experiment, the amount if freezing is with 70% reduced in the mutant compared to the wt, indicating that for the major part, amygdala-dependent fear conditioning is dependent on CAMKIIa, which should be the conclusion of the data. However, the authors now indeed mention that the amygdala-dependent cued fear conditioning is impaired throughout the manuscript. However, their final sentence in the significance statement and discussion needs to be revised: “Considering a prevalent role of CaMKIIa in different types of memory, possible involvement of a secondary CaMKIIa activity-independent mechanism in fear-related memory could lead to a novel treatment of abnormal fear memory-associated disorders, such as post-traumatic stress disorder”. Since the data shows that for the major part (75%) the memory is CaMKIIa activity-dependent, this sentence needs to be removed.

## Author Response

eN-TNWR-0133-18X

Differential involvement of kinase activity of Ca2+/calmodulin-dependent protein kinase II? in hippocampus- and amygdala-dependent memory revealed by kinase-dead knock-in mouse

Yoko Yamagata, Yuchio Yanagawa, Keiji Imoto

Point-by-point responses to the Reviewers' comments:

We appreciate very much the Editor's and Reviewers' valuable comments and suggestions. We have revised our manuscript accordingly. The following are point-by-point responses to the Reviewers' comments.

Synthesis of Reviews:

The authors studied hippocampus- and amygdala-dependent memory formation in a knockin mutant mouse line that blocks alphaCaMKII kinase activity. Studies with these knockin mutants are very important as the general alphaCaMKII knockout mice suffer from a compensatory translocation of betaCaMKII into the PSD, which does not appear to occur in these knockin mice. The literature on CaMKIIa and the involvement in learning and memory is already substantial. The fact that both hippocampal- as well as amygdala-dependent learning and memory is severely dependent on CaMKIIa-dependent activity is also already known as indicated by the authors themselves when citing Rodrigues et al., 2004 and Murakoshi et al., 2017. In addition, these 2 papers both show residual learning and memory present, which is similar to what the authors here show.

Response: In spite of the reviewer's indication, the two preceding papers (Rodrigues et al., 2004; Murakoshi et al., 2017) did not address residual learning and memory present after injection of a CaMKII inhibitor into the amygdala. Such injection studies always accompany a risk of incomplete blockade of CaMKII due to insufficient concentration and/or diffusion of the inhibitor, which could lead to observation of “residual” memory. Besides, they did not examine the effects of injection into the hippocampus, and thus, their studies did not compare the involvement of CaMKII activity in hippocampus- vs. amygdala-dependent memory.

On the other hand, we carefully examined the results from contextual and cued memory tests with and without kinase activity of CaMKII? by using the wild-type and kinase-dead CaMKII? (K42R) knock-in (KI) mice. Since kinase activity of CaMKII? is completely gone in the KI mouse, which is different from injection studies, it is reasonable to assume that the residual memory in the KI mouse is derived from something other than CaMKII? kinase activity, which is a novel finding as illustrated in Fig. 6. We already discussed these points in detail in the Discussion section (p. 42-43, lines 642-667).

In the current report, the authors found evidence that despite the lack of alphaCaMKII activity a long-lasting tone fear memory can be generated. In contrast, the mutants have substantial impairments in hippocampus-dependent spatial memory formation and contextual discrimination. Further, the mice seem to exhibit increased generalized fear. This leads to the conclusion that, amygdala-, but not hippocampus-dependent memory can be formed in absence of alphaCaMKII activity and that the kinase is required for establishing specific memories. This finding is important not only for understanding the molecular bases of memory storage, but also for developing treatments of PTSD. The work suggests that massed training can overcome a deficit in tone fear conditioning but not contextual fear conditioning. This is a different phenotype from the T286A mutants, which have normal pre-tone freezing; and other CaMKII mutants were not tested as extensively in fear conditioning. Thus, the result suggests that under strong fear stimulation generalized fear is generated; this is interesting as in PTSD fear is generalized. The authors should discuss this point in greater detail. In fact the authors may wish to quantify the amount of generalized freezing per animal. Is it possible that this enhanced generalized freezing is due to loss of input specificity of synaptic plasticity? It is important to substantiate this point because the mutants appear to be a great model of PTSD.

Response: According to the reviewer's comments, we included detailed discussion in the last two paragraphs in the Discussion section about (1) generalized fear response after intense training of fear conditioning in the CaMKII? (K42R)-KI mouse and (2) its relationship with post-traumatic stress disorder (PTSD) (p. 46-49, lines 702-750).

(1) We assume that two factors could account for fear generalization in the KI mouse, i.e., impaired contextual encoding in the hippocampus and impaired input-specificity of conditioning in the amygdala. Details are described in the second paragraph from the last in the Discussion section (p. 46-48, lines 702-734).

(2) Since generalized fear response disappeared when re-tested 4 w later in the KI mouse, extinction of increased fear memory seems to be preserved. Thus, the KI mouse does not fit as a PTSD model. Rather, we assume that the lack of kinase activity of CaMKII? could be one of the predispositions or risk factors toward the development of PTSD. Details are described in the last paragraph in the Discussion section (p. 48-49, lines 735-750).

(3) As suggested by the reviewer, quantifying the amount of generalized freezing per animal would be interesting, but the values would vary a lot depending on how similar the context and cued chambers are, on the number of repetition of CS-US conditioning, and on how to define “generalized freezing”. Aside from that, according to the reviewer's suggestion, we counted the number of animals that showed generalized freezing after 5CS-US conditioning (Fig. 4D), by using a tentative definition as follows: percent freezing time in the cued chamber without tone (Pre-tone) higher than both 40% and the values in the contextual chamber (Context) and in the cued chamber with tone (Tone) [i.e., Pre-tone freezing &gt; 40%, &gt; Context freezing, and &gt; Tone freezing]. According to this tentative definition, three out of 14 KI mice showed generalized freezing, whereas none out of 15 wild-type mice did. Thus, unlike wild-type mice, a certain number of KI mice actually showed generalized freezing, although the number itself was not so high under the current conditions. It would be of great interest to examine the effects of more intense training and of different types of cued chamber. However, these are out of scope of the current study and will be investigated in the future.

The amount of freezing behavior in the cued conditioning of the mutant compared to the wt is still significantly reduced in all the fear conditioning experiments performed. For example, in the 1CS-US experiment, the amount if freezing is with 70% reduced in the mutant compared to the wt, indicating that for the major part, amygdala-dependent fear conditioning is dependent on CAMKIIa, which should be the conclusion of the data. However, the authors now indeed mention that the amygdala-dependent cued fear conditioning is impaired throughout the manuscript. However, their final sentence in the significance statement and discussion needs to be revised: “Considering a prevalent role of CaMKIIa in different types of memory, possible involvement of a secondary CaMKIIa activity-independent mechanism in fear-related memory could lead to a novel treatment of abnormal fear memory-associated disorders, such as post-traumatic stress disorder”. Since the data shows that for the major part (75%) the memory is CaMKIIa activity-dependent, this sentence needs to be removed.

Response: According to the reviewer's comment, we revised the last sentence in the Significance Statement as follows: “The role of CaMKII? kinase activity in distinguishing different contexts indicates its possible involvement as a measure against abnormal fear memory-associated disorders, such as post-traumatic stress disorder.” (p. 6, lines 76-79).

We also revised the last sentence in the Discussion section as follows: “Further investigation of the kinase-dead CaMKII? (K42R)-KI mouse would lead to the understanding and novel therapeutics of PTSD and other abnormal fear memory-associated disorders.” (p. 48-49, lines 748-750).
